# Competitive food availability in schools before and after the onset of COVID-19: an interrupted time series analysis

**DOI:** 10.3389/fpubh.2026.1731287

**Published:** 2026-02-12

**Authors:** Sarah Martinelli, A. Bea Ronan, Francesco Acciai, Punam Ohri-Vachaspati

**Affiliations:** College of Health Solutions, Arizona State University, Phoenix, AZ, United States

**Keywords:** competitive foods, food environment, National School Lunch Program, school meals, universal free meals

## Abstract

**Purpose:**

School meal programs often offer competitive foods (CF), sold during lunch outside school meals and/or in vending machines. Although required to meet nutrition guidelines, CF are often less nutritious than school meals, and their presence is associated with lower meal participation. This study examines the prevalence of CF in public schools before and after COVID-19.

**Methods:**

Surveys examining the school food environment were conducted in public schools in 4 New Jersey cities, from school year (SY) 2014–15 to SY 2023–24. Prevalence of CF was assessed using an interrupted time series analysis, comparing pre- and post-COVID-19 years.

**Results:**

In 2021, CF availability was 31.5 percentage points lower than the expected level if the pre-COVID trend had continued (*p* < 0.001).

**Discussion:**

Findings suggest that COVID-related school nutrition policy changes, including Universal Fee Meals and increased reimbursements, likely reduced CF availability. Identifying strategies to limit CF could contribute to healthier school food environments.

## 1 Introduction

Each day, U.S. schools provide over 45 million meals to children through the National School Lunch Program and School Breakfast Program, which are subsidized by the U.S. Department of Agriculture (USDA) (1). These meals must follow USDA-mandated nutritional standards, which were updated in 2010 under the Healthy Hunger-Free Kids Act (HHFKA) and again in 2024 (2). Research has shown that school meals are the healthiest meals students consume in a day (3). Alongside USDA meal programs, many schools also offer competitive foods (CF) i.e., items sold à la carte in cafeterias, vending machines, and school stores. While CFs are regulated under the Smart Snacks in Schools standards introduced by the HHFKA, they are typically less nutritious than the USDA’s reimbursable meals (4, 5). Their availability is associated with lower school meal participation (6) and higher rates of increase in obesity in New Jersey schools (7). Evidence also suggests that stronger CF nutrition standards are associated with lower student intake of sugar and fat as well as healthier body mass index (BMI) outcomes (8–10). For instance, prior to the implementation of Smart Snacks, California implemented a similar policy, limiting the amount of sugar and fat allowed in CF served in its schools. Analyses of BMI trends pre- and post-policy implementation among California fifth graders showed improved overweight/obesity prevalence statewide, with sharper declines in higher-income areas (9). These findings suggest that policies impacting CF availability and nutrient quality may be a useful tool for combating childhood obesity.

Schools, however, often rely on CF to generate income, as USDA reimbursements for meals served do not always cover the full cost of meal preparation and distribution (11). Additionally, schools face limitations on adjusting meal prices, further constraining their ability to cover costs. During the COVID-19 pandemic, the USDA issued waivers that provided free meals to all students and increased reimbursement rates, leading to higher school meal participation and increased revenue, while potentially reducing schools’ reliance on CF. Moreover, supply chain disruptions during the pandemic likely further limited schools’ ability to offer typical CF options (12).

This study uses longitudinal data from four lower-income cities to assess whether and how the availability of CF in schools changed before and after the onset of the COVID-19 pandemic.

## 2 Materials and methods

This secondary analysis uses data from the New Jersey Child Health Study, which examines the impact of food and physical activity environments on children’s weight and health outcomes over time. Data on school food environments were collected through surveys from public schools located in four New Jersey cities: Camden, New Brunswick, Newark, and Trenton. The survey, adapted from prior research, included questions about foods and beverages offered (a) as part of reimbursable school lunches, (b) à la carte, and (c) in vending machines. A comprehensive list of food items available in vending machines and à la carte options can be found in Appendix Table 1.

The target population consisted of all K-12 public schools operating in one of the four study cities at each data collection point between 2012–13 and 2023–24. Surveys were distributed to school nurses in all eligible schools via Qualtrics^®^ (Provo, UT, United States) online platform or on paper. Each administration of the survey applied the questions to the current and preceding school year(s). In-service training and instructions for completing data collection were provided by study staff, coordinated by nursing divisions within each district. Nurses were instructed to consult with school food service staff to complete the food-related sections and were provided with a $50 gift card to incentivize completion of the survey. Since Smart Snacks guidelines were implemented in SY 2014–15, the current analysis is limited to data from SY 2014–15 to SY 2023–24. A full summary of survey development and study design is described elsewhere (13). This study was considered exempt by the Institutional Review Board of Arizona State University. The survey responses addressing the availability of CF in vending machines and à la carte during lunch were used to assess the presence of CF in each school. Data on school level (elementary vs. secondary schools), student enrollment, racial/ethnic proportions of students, and proportion of students eligible for Free and Reduced Price Meals (FRPM) were obtained from the National Center for Education Statistics (14). The proportion of students eligible for FRPM serves as a proxy for household income, as eligibility for school meals is determined by earning 185% or less of the federal poverty line (e.g., $32,150 for a family of 4 in SY 25–26). This metric is commonly used to approximate household income when direct income data are not available (15). School Community Eligibility Provision (CEP) participation status was obtained from the New Jersey Department of Agriculture; this variable was included as a covariate because previous research has shown that CEP schools are less likely to offer CF (6, 12).

All analyses were run using Stata 18 (StataCorp LLC, College Station, TX, United States, 2017). Multiple imputations by chained equations were used to account for item nonresponse. Descriptive statistics were used to examine all variables in the analysis. The outcome variable was presence of CF (yes/no); the two primary predictors were (1) a continuous time variable (in years), and (2) a binary time period indicator, defined as pre-COVID (from SY 2014–15 to SY 2019–20) and post-COVID onset (from SY 2021–22 to 2023–24). Data were available from 136 schools pre-COVID and 85 schools post-COVID onset. The number of schools surveyed ranged from 94 to 110 pre-COVID, and 47 to 85 post-COVID onset. This variation in sample size depended on schools’ opening or closing over the study period and unit nonresponse. Covariates included school CEP status (yes/no), the majority race/ethnicity of enrolled students (majority Black, majority Hispanic, or majority White/other race), the proportion of students qualifying for Free and Reduced-Priced Meals (categorized into tertiles), school level (elementary vs. secondary schools), and city.

An interrupted time series analysis within a logistic regression was used to model the presence of CF over time. An interaction term between the continuous time variable and the binary time period (pre- vs. post-COVID) indicator was used to estimate two separate linear trends, one for each time period. Postestimation commands (i.e., *margins* and *lincom*) were used (1) to test whether the pre- and post-COVID slopes were different from zero, (2) to test whether the two slopes differed from each other, and (3) whether there was a level change between the pre- and post-COVID prevalence. A subsequent logistic regression including year as a categorical variable provided yearly adjusted predicted prevalence estimates. Lastly, a logistic regression interacting the binary time period indicator variable with each covariate was used to assess pre- and post-COVID differences in predicted prevalence of CF overall and by school demographics. In all models, we included city fixed-effects and estimated cluster-robust standard errors at the school level, to obtain estimates that are robust to within-cluster dependence. Statistical significance was determined using an alpha level of 0.05.

## 3 Results

**Figure 1:**
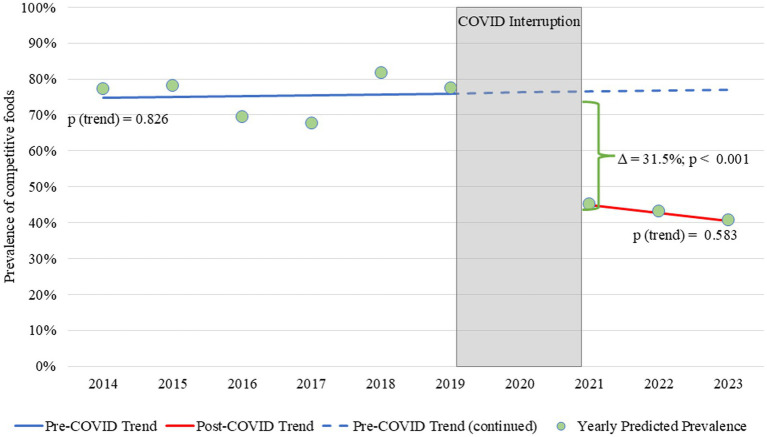
Trend in the prevalence of competitive foods before and after COVID-19-related school meal disruptions and policy changes. Pre- and post-COVID slopes were 0.003 and −0.023, respectively. Neither slope was significantly different from zero, and they were also not different from each other (∆χ^2^ = 0.026, *p* = 0.550). The bracket between the lines highlights the estimated level change in predicted CF prevalence in 2021.

Over the entire study period, the majority of study schools were elementary schools and had a predominantly Hispanic student population (Table 1). Figure 1 shows the predicted linear trend for CF prevalence from SY 2014–15 to SY 2019–20 and post-COVID onset from SY 2021–22 to 2023–24, along with the year-by-year predicted values. There was no difference in the slope between the pre- and post-COVID periods (*p* = 0.550), as both slopes were approximately flat. However, a significant downward level shift in CF availability occurred in SY 2020–21. Under the assumption that the pre-COVID trend would have continued if the COVID pandemic (i.e., the interruption in our time series) did not occur, we would have expected the prevalence of CF in 2021 to be 76.5%; instead, it was 45.0%, which corresponded to a 31.5 percentage point decline (*p* < 0.001). Pre-COVID, CF prevalence differed significantly between CEP and non-CEP schools and across school levels, with non-CEP schools and middle/high schools more likely to offer CF (Table 2). Post-COVID, CF prevalence decreased significantly across all demographic groups and no differences were observed by CEP status. However. CF levels remained higher in middle/high schools compared to elementary schools (59.0% vs. 34.5%, *p* < 0.001).

**Table 1:** New Jersey child health study sample demographics by year for school years 2014–15 to 2023–24 (*N* = 841).

School characteristics	2014 (*n* = 110)	2015 (*n* = 110)	2016 (*n* = 106)	2017 (*n* = 108)	2018 (*n* = 94)	2019 (*n* = 96)	2021 (*n* = 85)	2022 (*n* = 85)	2023 (*n* = 47)
	*n* (%)	*n* (%)	*n* (%)	*n* (%)	*n* (%)	*n* (%)	*n* (%)	*n* (%)	*n* (%)
Community eligibility provision participation									
Non-CEP	66 (60.0)	77 (70.0)	77 (72.6)	79 (73.2)	55 (58.5)	52 (54.2)	38 (44.7)	41 (48.2)	1 (2.1)
CEP	44 (40.0)	33 (30.0)	29 (27.4)	29 (26.9)	39 (41.5)	44 (45.8)	47 (55.3)	44 (51.8)	46 (97.9)
School level									
Elementary schools	74 (67.3)	74 (67.3)	71 (67.0)	73 (67.6)	62 (66.0)	64 (66.7)	55 (64.7)	55 (64.7)	31 (66.0)
Mid/high schools	36 (32.7)	36 (32.7)	35 (33.0)	35 (32.4)	32 (34.0)	32 (33.3)	30 (35.3)	30 (35.3)	16 (34.0)
School majority race based on enrolled students									
Majority Hispanic	54 (49.1)	55 (50.0)	58 (54.7)	62 (57.4)	52 (55.3)	57 (59.4)	53 (62.4)	54 (63.5)	37 (78.7)
Majority non-Hispanic black	50 (45.5)	52 (47.3)	45 (42.5)	43 (39.8)	40 (42.6)	36 (37.5)	31 (36.5)	30 (35.3)	10 (21.3)
Majority non-Hispanic white or mixed	6 (5.5)	3 (2.7)	3 (2.8)	3 (2.8)	2 (2.1)	3 (3.1)	1 (1.2)	1 (1.2)	0 (0.0)
Proportion of students qualifying for free and reduced-price meals									
Lower FRPM eligibility (25.2–77.4%)	18 (16.8)	51 (47.2)	44 (43.1)	37 (35.9)	47 (60.3)	33 (41.8)	38 (45.2)	35 (42.2)	28 (62.2)
Middle FRPM eligibility (77.5–87.5%)	42 (39.3)	31 (28.7)	34 (33.3)	32 (31.1)	26 (33.3)	32 (40.5)	39 (46.4)	39 (47.0)	13 (28.9)
Higher FRPM eligibility (87.6–97.3%)	47 (43.9)	26 (24.1)	24 (23.5)	34 (33.0)	5 (6.4)	14 (17.7)	7 (8.3)	9 (10.8)	4 (8.9)

CEP, community edibility provision; Majority race defined as 50% or more of enrolled students are from a given race/ethnicity.

**Table 2:** Predicted prevalence of competitive foods presence in New Jersey schools, overall and by school demographics, pre-COVID (2014–2019) and post-COVID (2021–2023).

School characteristics	Pre-COVID			Post-COVID			Pre–post difference
	*n*	Predicted Prevalence	*p* value	*n*	Predicted prevalence	*p* value	*p* value
Overall (all schools)	624	75.2%	–	217	42.2%	–	<0.001
Community eligibility provision participation							
Non-CEP	406	79.6%	0.027	80	50.0%	0.219	0.001
CEP	218	65.1%		137	35.1%		<0.001
School level							
Elementary schools	418	68.5%	<0.001	141	34.5%	0.006	<0.001
Mid/high schools	206	90.0%		76	59.0%		<0.001
School majority race based on enrolled students							
Majority Hispanic	266	75.0%	Ref	71	44.8%	Ref	<0.001
Majority non-Hispanic black	338	75.3%	0.958	144	38.0%	0.434	<0.001
Majority non-Hispanic white or mixed	20	79.1%	0.678	2	Not estimable	–	–
Proportion of students qualifying for free and reduced-price meals							
Lower FRPM eligibility	230	75.4%	Ref	101	36.7%	Ref	<0.001
Middle FRPM eligibility	197	73.3%	0.661	91	44.7%	0.369	<0.001
Higher FRPM eligibility	150	79.1%	0.461	20	51.6%	0.372	0.074

CEP, community edibility provision; Majority race defined as 50% or more of enrolled students are from a given race/ethnicity.

To assess the sensitivity of our findings, we conducted a series of robustness checks. First, we restricted the analysis to schools with observations in both the pre- and post-COVID periods (*n* = 615). Second, we conducted separate analyses to predict vending presence and á la carte presence separately (*n* = 841). In all cases, the findings were consistent with results from the full sample.

## 4 Discussion

Our findings show a marked decline in the prevalence of CF after the onset of COVID-19, suggesting that COVID-19-related disruptions and policy changes, such as the implementation of Universal Free Meals (UFM), increased meal reimbursement rates, and supply chain challenges, likely contributed to this reduction. While supply chain disruptions in the early stages of the pandemic likely played a significant role in the availability of CF, the availability of CF remained lower even after supply chain challenges improved. Further, CF offerings remained lower after UFM ended nationally in the fall of 2022, suggesting that schools retained these new practices once they were established. Prior research has shown that schools participating in CEP—i.e., those offering free meals to all students—are less likely to offer CF (6, 12). This supports the interpretation that the introduction of UFM in the early stages of the pandemic may have played a role in reducing CF availability.

Given that CF (1) are generally less healthy than USDA reimbursable meals offered through the National School Lunch Program (4, 5), (2) contribute to lower participation in such programs (6), and (3) were linked to higher prevalence of obesity in longitudinal research (7, 9), identifying policies that reduce schools’ reliance on CF is critical. Yet, schools often view CF as a necessary revenue source, with over 80% of school meal directors nationwide reporting that current USDA reimbursement rates do not fully cover meal production costs (11). To further reduce the availability of CF, strategies such as increasing reimbursement rates and supporting widespread participation in school meals through UFM programs such as CEP should be prioritized.

To our knowledge, no other studies have specifically examined changes in the availability of CF in schools post-COVID-19 either in the US or internationally. However, many countries have implemented policies aimed at limiting the availability of CF in schools and/or establishing minimum nutrition guidelines for these foods (16). While the presence of nutrition standards for CF has a positive impact on the availability of healthier foods in schools (17), the implementation and enforcement of these policies are often imperfect. As a result, on average, CF are still less healthy than the foods provided through school meal programs (4, 5). For example, in Australia, school canteens use a “stop light” classification system to set policies and what should be offered for sale. In this system, foods are categorized as either red, yellow, or green based on nutritional criteria, with red representing the least healthy options, which should not be offered for sale in school canteens. Despite this policy, nearly all schools (98.5%) offered at least one red category item (18). Similar patterns are observed in both Spain and Canada, where many foods sold in campus vending machines do not meet nutritional standards established in these countries (19, 20).

Many nations are concerned about the nutritional quality of foods sold to students outside of school meals and regulatory efforts targeting CF are not always successful. In contrast, countries with long-standing universal free meal programs, such as Sweden, Finland, and Estonia generally do not offer CF (16, 21, 22). In these school systems, meals and snacks are provided at no charge and are required to meet the same nutrition standards, including snacks offered to students in before and after school programs. This complete support eliminates the demand from students to buy CF and, without a market for them or a financial need for the school, these products are simply not offered. Implementing comprehensive UFM programs may therefore be more effective at reducing the prevalence of CF in schools than regulations that target these foods in isolation.

While this study has several strengths, it also has limitations. A key strength of this study is its longitudinal design. The availability of data from the same group of schools over nearly 10 years allowed for consistent and comparable assessment of trends over time. The three time points available in the post-COVID period is both a strength and a limitation; while these data enable the identification of the post-pandemic trend, additional follow-up would be necessary to more fully characterize its long-term trajectory. This study also has limitations related to generalizability, as this sample is drawn from low-income, high-minority urban schools within a single state. Finally, we did not have access to data specifically measuring school food supply chain status, which limits our ability to assess the extent to which supply chain disruptions contributed to the observed changes in CF availability.

## 5 Conclusion

School food environments play a critical role in supporting students’ access to healthy, nutritious foods during the school day. Because school meals tend to be healthier than CF, and the presence of CF has been associated with lower school meal participation, reducing the availability of CF in schools represents one potential strategy for improving the nutritional quality of foods accessible to students. While external factors, such as the supply chain disruptions experienced during the COVID-19 pandemic, may have a short-term impact on the availability of CF, long-term strategies are needed to more comprehensively address their presence in schools. One potential method for lowering the prevalence of CF in schools is to ensure sustainable funding for school meal programs through adequate federal reimbursements, alongside increased support for UFM programs such as CEP. These steps may enhance the financial sustainability of school meal programs while fostering healthier school food environments by reducing reliance on CF sales.

## Data Availability

The data analyzed in this study is subject to the following licenses/restrictions: This study is a secondary data analysis. Datasets include data on a wide variety of variables beyond the scope of this paper. Requests to access these datasets should be directed to SM, Sarah.Martinelli@asu.edu.
