# Supplementary material for: Competitive food availability in schools before and after the onset of COVID-19: an interrupted time series analysis
Source: Front Public Health. 2026 Feb 12;14:1731287. doi: 10.3389/fpubh.2026.1731287 (PMC12935626; doi:10.3389/fpubh.2026.1731287)
Supplement: Supplementary file 1 [file Table_1.docx]

| **Appendix Table 1. List of competitive food items in K-12 schools in New Jersey** | | |
| --- | --- | --- |
| **Vending Machine Items** | **A la carte Items** | |
| Bottled Water | Bottled Water | Dairy Foods, Lower in Fat |
| 100% Juice | 100% Juice | Raw fruit & vegetables |
| Unflavored Milk (Fat-free / 1%) | Unflavored Milk (Fat-free / 1%) | Salad Bar |
| Flavored Milk (Fat-free) | Flavored Milk (Fat-free) | Sandwiches |
| Unflavored Milk (Full fat / 2%) | Unflavored Milk (Full fat / 2%) | Fried Potatoes |
| Flavored Milk (Full fat / 2% / 1%) | Flavored Milk (Full fat / 2% / 1%) | Pizza |
| Juice Drink | Juice Drinks | Frozen Desserts |
| Soda, Diet Soda | Soda, Diet Soda |  |
| Energy or Sports Drinks | Energy or Sports Drinks |  |
| Salty snacks | Salty Snacks |  |
| Cookies, cakes | Cookies, Cakes |  |
| Candy | Candy |  |
